# An Assessment of the Ratio between Upper Body Push and Pull Strength in Female and Male Elite Swedish Track and Field Throwers

**DOI:** 10.3390/sports12080201

**Published:** 2024-07-24

**Authors:** Jesper Augustsson, Ted Gunhamn, Håkan Andersson

**Affiliations:** 1 Department of Sport Science, Faculty of Social Sciences, Linnaeus University, 39182 Kalmar, Sweden; ted.olssongunhamn@lnu.se; 2 High Performance Center, Strength and Conditioning Institute, 35246 Vaxjo, Sweden; hakan.andersson@hpcsweden.com

**Keywords:** static, test–retest reliability, force, muscle balance

## Abstract

Data on the strength ratio between agonist and antagonist muscles are frequently examined in sports testing, given its correlation with athletic performance. The purpose of this study was to determine the agonist-to-antagonist ratio of upper body strength in female and male elite Swedish track and field throwers using a new push (bench press) and pull (supine bench row) test device, and to determine its reliability. The study involved eight female and nine male athletes, aged 19–29 years, engaging, respectively, in discus, hammer, and shot put competitions at both national and international levels. The athletes’ maximum isometric force was assessed during the bench press (push) and supine bench row (pull) exercises, respectively, using a custom-built test device. The test–retest reliability of the device was also examined. The total push-to-pull strength ratio for the female throwers was 1.15, whereas male throwers demonstrated a ratio of 1.22. Total push and pull force for the female throwers was significantly less than for the male throwers (5511 N vs. 8970 N, *p* < 0.001). Intraclass correlation coefficients ranged from 0.93 to 0.96 for the bench press and supine bench row exercise, indicating that the push and pull test device was highly reliable. The main findings of this study were that elite female and male discus, hammer, and shot put throwers exhibited 15% and 22% more pushing (bench press) than pulling (supine bench row) strength. Push and pull strength in the female throwers ranged from 47% to 71% of that of the male throwers. The push and pull test device is a reliable tool in establishing the agonist-to-antagonist ratio of upper body strength of athletes. Coaches and athletes may benefit from examining upper body push and pull strength ratios for training planning and prescription.

## 1. Introduction

Power and strength are considered essential for the execution of numerous athletic activities [1,2]. Consequently, many athletes and coaches prioritize the development of strength and power [3]. Further, it seems that increasing muscular strength may have no equivalent substitute in enhancing an individual’s performance across various general and sport-specific skills, while also concurrently lowering the risk of injury during the execution of these skills [4]. Athletic strength and power relate to the forces or torques generated in sports-related activities [5]. Assessing them serves various purposes, including diagnosing strength, identifying talent, monitoring the effects of training, injury prevention or rehabilitation interventions, and gauging the significance of strength and power in athletic pursuits [6].

Information regarding the strength ratio between agonist and antagonist muscles is commonly analyzed in sports testing due to its association with athletic performance [7] as well as its correlation to sports injuries [8,9]. Within the literature, researchers have investigated opposing muscle groups and determined strength ratios in different athletic populations using isokinetic, isometric and isotonic testing. For the upper extremity, the agonist-to-antagonist ratio of strength has been examined for the shoulder joint (abduction/adduction and internal/external rotation) [10,11] and elbow joint (flexion/extension) [12]. In studies focused on the upper extremity, the most explored strength ratio is internal to external rotation of the shoulder [13,14,15,16].

Currently, there is no gold standard in the literature to assess the ratio between upper body push and pull strength. Moreover, no push and pull test method assesses strength in an identical test position, the way hamstring-to-quadriceps strength ratios are compared using seated knee extension and flexion, for example. Therefore, developing a device where the push and pull tests precisely mirror each other, using identical positioning for both movements, may be of interest.

Track and field throwing disciplines such as discus, hammer, and shot put are high-intensity activities that demand intricate technical skills and rapid force generation [17]. Despite their distinct characteristics, athletes across these events dedicate significant time to resistance training, aiming to increase strength and power. Exercises like the bench press and bench row are fundamental components in the training regimens of elite track and field throwers. However, to the best of our knowledge, the strength ratios between these exercises have not been investigated for these athletes.

Taken together, for athletes with a need for high levels of upper body strength, such as elite track and field throwers, we believe that measuring both push and pull strength and determining their ratio is highly relevant. This understanding of upper body push and pull strength ratios can be beneficial for athletes and coaches in developing programming strategies that encompass monitoring training effects, performance outcomes, and injury prevention. Moreover, developing a method where pushing and pulling tests are conducted in identical positions in a single device could prove valuable for upper body strength ratio assessment.

The purpose of this study was to determine the agonist-to-antagonist ratio of upper body strength in female and male elite Swedish track and field throwers using a new push (bench press) and pull (supine bench row) test device, and to determine its reliability.

## 2. Materials and Methods

### 2.1. Trial Design and Experimental Approach

The study employed a cross-sectional design, wherein testing for each participant was conducted within a single test session. A custom device, specifically designed for this study, was utilized to assess maximal isometric force during a bench press and a supine bench pull exercise, respectively. This approach aimed to determine the push and pull strength ratio in elite Swedish track and field throwers.

### 2.2. Development of the Test Device

**Figure 1:**
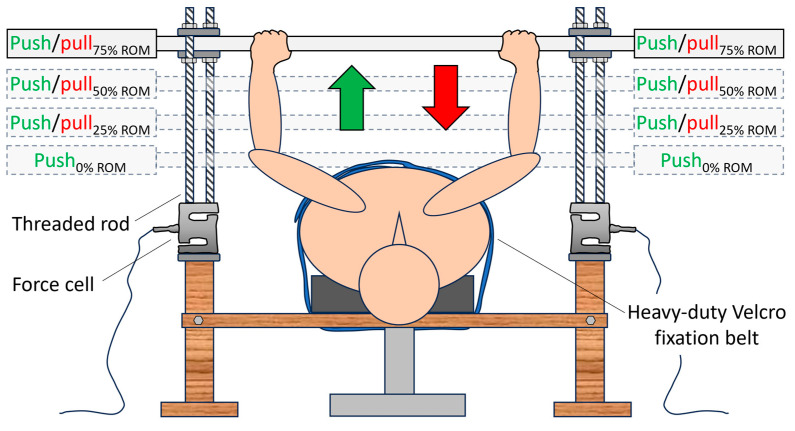
A schematic illustration of the apparatus used for measuring push (bench press) and pull (supine bench row) strength ratios. Dual, bi-directional (tension and compression) load cells were employed to collect the participants’ maximal isometric forces for both the right and left sides. Force data were collected with the barbell at the chest (push only) and at the 25%, 50%, and 75% position of the full range of motion (ROM) for each exercise/motion (bench press–supine bench row/push–pull).

We have developed a push and pull test device to assess the agonist-to-antagonist ratio of upper body strength. The device isometrically measures both bench press and bench row performance in an identical test position, with the participant lying supine on a training bench. The concept of the device is based on the capacity of bi-directional load cells to measure both tension (push) and compression (pull) forces. A schematic illustration of the apparatus for simultaneously testing push (bench press) and pull (supine bench pull) strength ratios is provided, using load cells (MuscleLab, Ergotest Technology AS, Langesund, Norway) for the right and left sides; see Figure 1.

**Figure 2:**
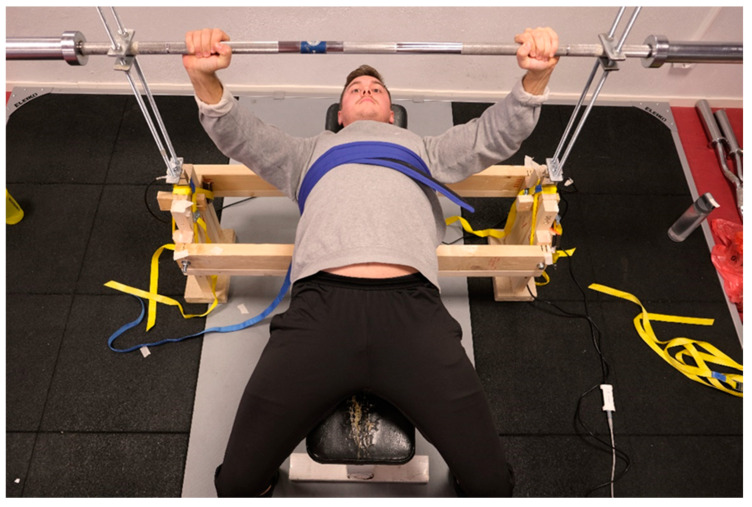
Testing set-up. The participant’s position during push (bench press) and pull (supine bench row) testing are illustrated. During supine bench row testing, the upper body was firmly secured to the bench by a heavy-duty Velcro fixation belt.

The apparatus consisted of horizontal wood studs that were anchored to the underside of a regular training bench. The studs were in turn connected to two vertical wood pillars onto which one load cell and two threaded rods (thread size M12) were placed on each side. An Olympic 20 kg barbell (Eleiko, Halmstad, Sweden) was placed between the threaded rods, secured over and under with 8 mm joint brackets and nuts. This construction allowed the barbell to be positioned at any distance from the participant’s chest with great precision, simply by screwing the nuts up or down the threaded rods. Once a particular position was set and the nuts were tightened, the device exhibited excellent mechanical rigidity, effectively restraining/minimizing joint movement. During supine bench row testing, the upper body was firmly secured to the bench by a heavy-duty Velcro fixation belt (Kajs belt, Medema, Kista, Sweden). The grip width was standardized to 81 cm during both pushing and pulling for all participants. The data collection for the load cells utilized a sampling rate of 200 Hz. Information from the load cells was synchronized through the MuscleLab system (V10.21, Ergotest Technology AS, Langesund, Norway). The software allowed collection of force data from each load cell separately as well as the sum of force from both load cells during testing. Before testing, the load cells were calibrated according to the operator’s manual procedure (Ergotest Technology AS, Langesund, Norway). The testing setup and the participant’s position during push (bench press) and pull (supine bench row) testing are illustrated in Figure 2.

### 2.3. Participants

#### 2.3.1. Elite Throwers and Their Upper Body Strength Ratios

Seventeen elite Swedish track and field throwers, including eight females, aged 19–29 years, participated in this study, engaging, respectively, in discus, hammer, and shot put competitions at both national and international levels (Table 1). To be included, the participants had to be highly trained athletes who were familiar with the isotonic bench press and bench row exercises. Participants with upper body injuries within the past six months were excluded from the study. Prior to testing, the participants were informed that they would perform a test of upper body strength; however, none of the participants were acquainted with or had executed isometric bench press or supine bench row.

#### 2.3.2. Sport Science Students Involved in the Reliability Tests of the Device

To examine the test–retest reliability of the device, 12 male sport science students (mean and SD age, height, body mass and strength training experience: 25 ± 3 years, 182 ± 5 cm, 84 ± 6 kg, and 8 ± 3 years, respectively) performed the push and pull test twice, five to seven days apart. Before being included in the study, all participants were informed of the risks and benefits of participation before providing written informed consent. Approval for this study was received from the Swedish Ethical Review Authority.

### 2.4. Procedures

The participants engaged in a general 5 min upper body warm-up which included arm and shoulder rolls and open arm criss-cross exercise. They then lay down on the bench and assumed bench press position while grabbing a barbell with fully extended arms, using a standardized 81 cm grip width. At that position, a test leader measured the vertical distance between the barbell and the chest, i.e., the range of motion (ROM), using a measuring stick. Next, two isometric pushing and pulling warm-up trials at 50% effort were also performed by each participant. Maximal isometric force data were then collected with the barbell at the chest (push only) and at the 25%, 50%, and 75% position of the full ROM for each exercise/motion (bench press–supine bench/push–pull). More specifically, the full ROM was divided by four to represent the total range of motion (e.g., if a participant’s full ROM was 40 cm, this value was divided by 4 = 10 cm increments). Participants would, in a random order, perform in total seven maximal isometric push and pull trials for three seconds in, if full ROM was, for example, 40 cm, 10 cm steps. At a distance of 0 cm from the chest, only pushing was performed. This is because, when pulling at this distance, the barbell is pressed into the chest, and the load cells (using the present set-up) do not register any data. For the next trial, the test leaders would raise the barbell (in this example, to 10 cm distance from the chest) by screwing the nuts up the threaded rods. Participants would then push and pull at 25% of the full ROM. Next, the test leaders would once again raise the barbell (in this case, to a 20 cm distance from the chest), and the participants would push and pull at 50% of the full ROM. Finally, the test leaders would raise the barbell (in this example, to a 30 cm distance from the chest), and the participants would push and pull at 75% of the full ROM. The rest period between trials at the four different distances was two minutes. Each push and pull attempt at a particular distance was separated by a one-minute rest period. In most cases, given the participants’ experience with strength training and the technical simplicity of the tests, only one trial per test and position was conducted. The instructions and verbal cues given to the participants were standardized to maintain consistency during the testing process. A sports physical therapist, possessing more than 25 years of experience in strength testing and training, supervised all testing and trial performances.

### 2.5. Test–Retest Reliability Procedures

The elite Swedish athletes who participated in the study were tested during a national team meeting for track and field throwing events, and it was only possible to perform the tests at this one instance. As mentioned in Section 2.3, Participants, to examine the test–retest reliability of the device, 12 male sport science students therefore performed the push and pull test twice, five to seven days apart. For this part of the study, the participants performed the test of pushing (bench press) and pulling (supine bench row) strength at either the 25%, 50% or 75% position of the full ROM. The participants were randomized to a particular position of the full ROM, employing a function for generating uniformly distributed random numbers (Excel for Microsoft 365 MSO, Microsoft Corporation, Redmond, WA, USA), with each position being performed by four participants.

### 2.6. Statistical Analyses

Data were analyzed using IBM SPSS Statistics (version 29, IBM, Armonk, NY, USA). The data under examination were found to be normally distributed based on the results of a Shapiro–Wilk test, allowing for the use of parametric tests for significance (*p* > 0.05). The results are presented as mean with SD. To detect differences in each position of the ROM for the isometric bench press and supine bench row tests between female and male throwers, a two-way analysis of variance (ANOVA) [2 (gender) × 7 (type of test)] was conducted. Post-hoc comparisons were performed using the Bonferroni method. Side-to-side differences in isometric bench press and bench row force between the dominant and non-dominant arms for female and male throwers were assessed with a paired samples T-test. The push-to-pull strength ratio in female and male throwers was calculated by dividing the mean value of the bench press test by the mean value of the bench row test. To describe the strength curves for the bench press and bench row exercises, mean isometric force data at different points in the full ROM—specifically, at the 0%, 25%, 50%, and 75% position of the full ROM—were plotted. To assess the test–retest reliability of the push and pull test device, intraclass correlation coefficients (ICC) [18] were calculated using the two-way random effects model for measurements, along with a 95% confidence interval (CI). The ICC values were categorized as follows: greater than 0.90 indicating high reliability, between 0.80 to 0.89 denoting good reliability, between 0.70 to 0.79 representing fair reliability, and values less than 0.69 indicating poor reliability [19]. Additionally, within-subject variation was determined by calculating the typical error expressed as a coefficient of variation (CV) [20]. The significance levels for analyses were set at *p* < 0.05.

## 3. Results

The total push-to-pull strength ratio for the female throwers was 1.15, whereas male throwers demonstrated a ratio of 1.22. The total push and pull force (i.e., the combined score at 25%, 50% and 75% position of the full ROM) for the female throwers was significantly less than that of the male throwers (5511 N vs. 8970 N, *p* < 0.001). Push and pull strength in the female throwers ranged from 47% to 71% of that of the male throwers.

A two-way ANOVA was performed to evaluate the effects of gender and type of test on isometric force. The results revealed a significant main effect concerning gender (F = 117.24; *p* < 0.001); a significant main effect regarding the type of test (F = 14.24; *p* < 0.001); and no significant interaction between gender and the type of test (F = 1.43; *p* = 0.211). Post hoc testing using the Bonferroni method indicated that, for females, pushing force at the 75% position of the full ROM was significantly higher than pushing force at 0% and 25% of ROM and pulling force at 25 and 50% of ROM (*p* < 0.05). For males, pushing force at the 75% position of the full ROM was significantly higher than both pushing and pulling force at all other ROMs (*p* < 0.05). Further, pulling force at 25% of the full ROM was significantly (*p* < 0.05) lower than pushing and pulling force at all other ROMs, except at pulling at 50% of ROM. For an account of the push and pull forces and strength ratios for all the different ROMs, see Table 2 and Table 3.

Side-to-side differences in isometric bench press and supine bench row force between dominant and non-dominant arms for the female and male throwers ranged 1–21%. For female throwers, significant side-to-side differences were observed in all positions of the bench press test (*p* < 0.05). Among male throwers, significant side-to-side differences were found in the bench press and supine bench row tests at the 75% ROM position (*p* < 0.05).

**Figure 3:**
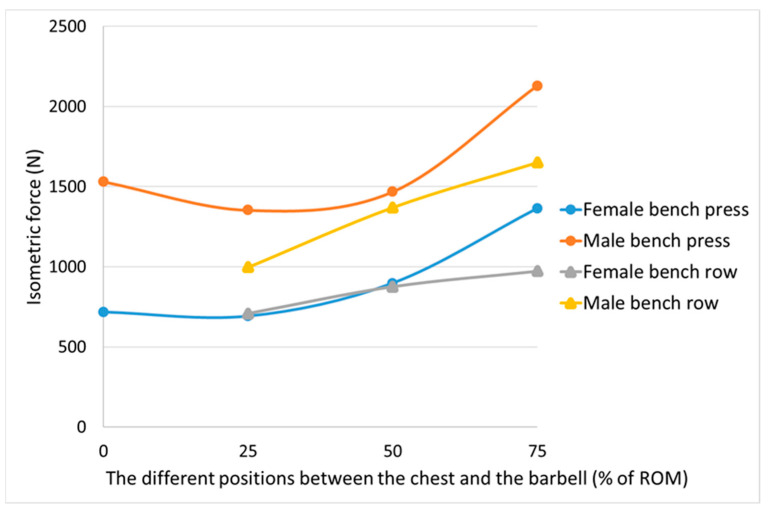
Mean isometric force data at different positions that illustrate the female (*n* = 8) and the male (*n* = 9) elite throwers’ strength curves for the bench press and the supine bench row exercises.

The participants’ strength curves for the bench press and the supine bench row exercises are depicted in Figure 3.

No significant test–retest differences were observed in either the bench press test or the supine bench row test. Mean ± SD isometric bench press force for test 1 was 1123 N ± 220 vs. 1147 N ± 298, for test 2 (*p* = 0.424). For the isometric supine bench row test, mean ± SD force for test 1 was 1008 N ± 215 vs. 1009 N ± 193, for test 2 (*p* = 0.962). The ICCs ranged from 0.93 to 0.96, the CI ranged 0.78–0.99, and the CV ranged from 3.32% to 3.54% for the isometric bench press and supine bench row tests.

## 4. Discussion

### 4.1. Ratio between Upper Body Push and Pull Strength in Female and Male Elite Swedish Track and Field Throwers

For athletes requiring substantial upper body strength, like elite track and field throwers, we argue that assessing both pushing and pulling strength and evaluating their ratio may hold significant importance. Understanding the relationship between upper body pushing and pulling strength may be beneficial for athletes and coaches in developing training strategies. This knowledge could potentially aid in monitoring training effects, assessing performance outcomes, and contributing to injury prevention efforts. When isometric force data were collected at 50% ROM, representing a “neutral” midpoint position, the push and pull strength ratios were very similar for both females and males, at 1.03 and 1.07, respectively. This suggests that the 50% ROM position may best reflect push and pull strength ratios and should perhaps be used if only one ROM, instead of all three, were to be tested.

To the best of our knowledge, very few studies have examined the ratio of upper body push and pull strength using the bench press and the bench row exercises. This is conceptually significant, given that the bench row test is more inherently antagonistic to the bench press test than a pull-up test. Furthermore, in our study, we introduce the isometric supine bench row test/exercise, a method that precisely mirrors the isometric bench press test. Additionally, our study specifically studies upper body strength ratios in female and male elite (i.e., successful) track and field throwers. Consequently, this research presents the first set of data showing the push and pull strength ratios within this athlete group.

Several studies have noted the existence of a ‘sticking region’ in both isotonic and isometric bench press exercises, a position where the capacity to generate force is diminished [21,22]. The bench press sticking region has been reported to occur at a mean distance of 13 cm [23] and 16 cm [24], respectively, between the chest and the barbell. This aligns with our results, where less bench press force was produced at 25% of the ROM compared to both the 0% and 50% positions, for both female and male throwers (see Figure 3). This indicates the presence of a sticking region approximately when the distance between the barbell and the chest was 10 cm. However, it is important to note that these differences in force within our relatively small sample were not statistically significant.

For the comparison of isometric strength levels for the bench press and supine bench row, the 25%, 50%, and 75% positions of the two exercises were chosen to represent the full ROM. The bench press exercise exhibited an ascending strength curve, meaning it was possible to produce more force during the last part of the ROM. In contrast, the supine bench row exercise displayed a descending strength curve, indicating that more force could be generated during the first part of the ROM. At the midpoint (50%) of the full ROM, a neutral position, the strength ratios for the push (bench press) and the pull (supine bench row) exercises (1.03 for females and 1.07 for males) demonstrated a well-balanced relationship between agonist and antagonist muscles (see Table 3). From a practical standpoint, if testing only one distance between the chest and the barbell, it may therefore be most valid and representative to evaluate the ratio of agonist-to-antagonist upper body strength at the midpoint of the full ROM.

Push and pull strength in the female throwers ranged from 47% to 71% of that of the male throwers. This is consistent with existing literature on sex differences in muscle strength, where female upper body muscle strength is typically reported to be around 50–60% of male upper body strength [25]. Additionally, within the athlete population, a notable difference in upper body muscle strength has been observed, with males demonstrating significantly greater strength than females [26].

Side-to-side differences in isometric bench press and bench row force between dominant and non-dominant arms for the female and male throwers ranged 1–21%. All participants were right-hand dominant; however, in five out of seven tests for females and four out of seven tests for males, forces were higher on the left side. This aligns with prior research on maximal strength asymmetries between the upper limbs [27], where participants were categorized into three groups: stronger on the non-dominant side, symmetric strength, and stronger on the dominant side, with percentages of 41%, 41%, and 18%, respectively.

### 4.2. The Development and Reliability of the Device Used to Measure Isometric Bench Press and Supine Bench Row Strength

The device used in this study enables direct comparisons of isometric strength levels for both the bench press and bench row exercises, maintaining an identical test position where participants lie supine on a training bench. The device operates on the principle of dual-purpose, bi-directional load cells, enabling the simultaneous measurement of both tension (push) and compression (pull) forces. This is, to our knowledge, the first agonist-to-antagonist upper body strength test device that is built upon this idea. It would seem possible to apply this approach to explore strength ratios for various opposing muscle groups in both the lower and upper extremities, other than upper body push and pull strength.

The ICCs varied between 0.93 to 0.96, the CI ranged 0.78–0.99, and the CV spanned from 3.32% to 3.54% for the isometric bench press and supine bench row tests. Notably, these results were achieved without participants undergoing any familiarization sessions. This affirms the high reliability of the push and pull test device and is in agreement with the high test–retest reliability noted in other isometric strength assessment such as, for example, the isometric mid-thigh pull test [28] and knee flexion [29] and knee extension strength [30]. The generally high reliability of maximal isometric testing noted in the literature is likely attributed to the absence of movement and often minimal skill/technique requirements. This allows participants undergoing the test to concentrate their efforts on generating maximal force, contributing to the overall reliability of the assessment.

### 4.3. The Study’s Limitations

The current study has certain limitations. The findings may have limited generalizability, as the study focuses on female and male elite track and field throwers. Furthermore, the sample size may be considered relatively small, and making comparisons across the different throwing events (discus, hammer, and shot put) was not deemed very meaningful. However, it is worth noting that this participant group does represent the majority of the population of elite female and male track and field throwers in Sweden. The current study’s cross-sectional approach captures strength ratios at one specific moment. To gain a more complete understanding of how these ratios change over time, a longitudinal study would be beneficial. Such an approach would allow researchers to track how factors like training, competition, and recovery periods affect the ratios. By following athletes for an extended duration, we could better grasp the long-term effects of strength training on push–pull ratios and overall performance. Our study relied solely on isometric strength tests. Although these provide useful data, they do not entirely reflect the dynamic aspects of sports performance. Adding dynamic assessments, such as isoinertial or isokinetic tests, would offer a more comprehensive view of athletes’ different strength qualities and their effects on performance. Such an approach could also reveal any differences between an athlete’s isometric and dynamic strength abilities. While our study brings up the possible link between strength ratios and injury risk, it does not explore this connection in depth. Future investigations should include a detailed analysis of athletes’ injury history and correlate it with strength ratios. Such research could lead to more specific and evidence-based recommendations for preventing injuries by addressing particular strength imbalances. Both neuromuscular and biomechanical factors contribute to strength. Future studies may incorporate electromyographic analysis to understand muscle activation patterns during push and pull exercises. Additionally, biomechanical assessments could reveal how joint angles, limb lengths, and movement mechanics influence strength ratios, providing a more holistic understanding of the factors at play. Concerning the test–retest reliability procedures, participants underwent strength testing for pushing (bench press) and pulling (supine bench row) at either the 25%, 50% or 75% position of the full ROM, rather than at all positions of the full ROM. Performing the push and pull test at all positions of the full ROM, we believe, would not have adversely affected the test–retest reliability results. Furthermore, while we were pleased to have the opportunity to assess the push and pull strength of Sweden’s top track and field throwers, practical constraints prevented us from conducting test–retest measurements directly with these elite participants. Instead, we included a test–retest component in our study using 12 strength-trained sport science students, who, in the context of a reliability study on a bench press and bench row test device, could be considered an adequately sized sample of highly suitable participants. This is the same approach that, for example, Opar et al. [31] used when developing a device for measuring knee flexor force in athletes. They used sub-elite athletes for the test–retest reliability part of the study, whereas professional elite athletes participated on a single occasion to assess the ability of the device to detect strength deficits in previously injured athletes. Further, we did not investigate the validity of the bench press and bench row, as these tests have demonstrated validity for assessing push and pull strength in athletes, as evidenced by studies such as Lum et al. [32] and Pineda et al. [33]. In fact, the bench press and bench row are well-established exercises, often used to assess the concurrent validity of new upper body strength tests [34,35]. Moreover, coaches and researchers in the sports field have utilized these tests for many decades. Nevertheless, although the test device showed high reliability, further validation against established methods of measuring dynamic and isometric strength would be beneficial. This would establish the device’s accuracy and consistency across different populations and settings. Taken together, while this study presents significant advancements in assessing upper body strength ratios in elite athletes, it is constrained by its limited sample size, cross-sectional design, and lack of dynamic testing.

### 4.4. Regarding Further Research

In the context of future research, the push and pull test device is designed for assessing upper body strength ratios. It can be employed to reliably generate a profile of physiological characteristics related to maximal strength, needed for competitive success in various athletic disciplines. These include, among others, track and field throwing events, handball, rugby, American football, wrestling, swimming, ice hockey, and volleyball. This broader applicability could help in developing guidelines for sport-specific strength training programs to assist coaches and athletes in optimizing their training approaches. These guidelines should address how to balance push and pull strength, periodize training to maintain ideal ratios, and implement corrective exercises for any identified imbalances. The push and pull test device may also be used to characterize the physical fitness capacity of other populations such as police officers, fire fighters, and various military personnel. Conclusively, as mentioned earlier, the sample size was not large enough to facilitate meaningful comparisons across elite athletes from the various throwing events (discus, hammer, and shot put). Nevertheless, exploring distinct patterns within each discipline remains of interest. For instance, there is a belief, at least anecdotally, that hammer throwers excel more as pullers than pushers. Collaboration among researchers from different countries could make such an investigation feasible, ensuring a sufficient number of elite athletes for meaningful comparisons between the various throwing events.

## 5. Conclusions

The main findings of this study indicate that female and male elite discus, hammer, and shot put throwers exhibited 15% and 22% more total (all ROMs combined) pushing (bench press) than pulling (supine bench row) strength, respectively. However, at the neutral, midpoint position (50% of the full ROM), the strength ratios for the push (bench press) and the pull (supine bench row) exercises (1.03 for females and 1.07 for males) demonstrated a well-balanced relationship between agonist and antagonist muscles. Push and pull strength in the female throwers ranged from 47% to 71% of that of the male throwers. For athletes and coaches, the clinical push and pull test device provides a reliable method to determine the ratio of strength between opposing muscle groups in the upper body.

## Figures and Tables

**Table 1:** Characteristics of participants (*n* = 17).

Characteristics *	*n*	Mean ± SD
Sex	8 (9)	
Discus	3 (3)	
Hammer throw	4 (4)	
Shot put	1 (2)	
Right hand dominant	8 (9)	
Age, year		24 ± 4 (22 ± 3)
Height, cm		178 ± 7 (188 ± 6)
Body mass, kg		81 ± 9 (108 ± 9)
Practice, hours per week		16 ± 5 (18 ± 4)
Distance between chest–barbell with fully extended arms (cm)		41 ± 3 (40 ± 4 cm)

* Data for female throwers are presented without brackets; data for male throwers are shown in brackets.

**Table 2:** Mean and SD push and pull strength in elite Swedish female (*n* = 8) and male (*n* = 9) track and field throwers. Isometric force (N) data were collected at the 25%, 50%, and 75% position of the full range of movement (ROM) for each exercise/motion (bench press–supine bench/push-pull).

Test	0% ROM	25% ROM	50% ROM	75% ROM
Bench press (N), females	718 ± 81	692 ± 105	898 ± 174	1364 ± 308 *
Supine bench row (N), females	N/A	708 ± 119	876 ± 135	973 ± 146
Bench press (N), males	1531 ± 439	1353 ± 394	1468 ± 373	2129 ± 687 #
Supine bench row (N), males	N/A	998 ± 140 §	1370 ± 180	1651 ± 232

* Indicates significant difference from pushing force at 0% and 25% of ROM and pulling force at 25 and 50% of ROM in females at a *p* < 0.05 level. # Indicates significant difference from both pushing and pulling force at all other ROMs in males at a *p* < 0.05 level. § Indicates significant difference from pushing and pulling force at all other ROMs, except at pulling at 50% of ROM in males at a *p* < 0.05 level. N/A (not applicable).

**Table 3:** Mean and SD push and pull ratios of upper body strength in elite Swedish female (*n* = 8) and male (*n* = 9) track and field throwers. Isometric force data were collected at the 25%, 50%, and 75% position of the full range of movement (ROM) for each exercise/motion (bench press–supine bench row/push-pull).

Test	25% ROM	50% ROM	75% ROM	All ROMs Combined
Bench press–supine bench row ratio, females	0.99 ± 0.12	1.03 ± 0.18	1.41 ± 0.34	1.15 ± 0.26
Bench press–supine bench row ratio, males	1.38 ± 0.47	1.07 ± 0.25	1.27 ± 0.28	1.22 ± 0.20

## Data Availability

Upon request, the corresponding author can provide access to the data presented in this study.
